# A-Syn(ful) MAM: A Fresh Perspective on a Converging Domain in Parkinson’s Disease

**DOI:** 10.3390/ijms25126525

**Published:** 2024-06-13

**Authors:** Peter A. Barbuti

**Affiliations:** Department of Neurology, Columbia University Irving Medical Center, New York, NY 10032, USA; pab2184@cumc.columbia.edu

**Keywords:** alpha-synuclein (αSyn), mitochondrial-associated ER membranes (MAMs), Parkinson’s disease (PD)

## Abstract

Parkinson’s disease (PD) is a disease of an unknown origin. Despite that, decades of research have provided considerable evidence that alpha-synuclein (αSyn) is central to the pathogenesis of disease. Mitochondria-associated endoplasmic reticulum (ER) membranes (MAMs) are functional domains formed at contact sites between the ER and mitochondria, with a well-established function of MAMs being the control of lipid homeostasis within the cell. Additionally, there are numerous proteins localized or enriched at MAMs that have regulatory roles in several different molecular signaling pathways required for cellular homeostasis, such as autophagy and neuroinflammation. Alterations in several of these signaling pathways that are functionally associated with MAMs are found in PD. Taken together with studies that find αSyn localized at MAMs, this has implicated MAM (dys)function as a converging domain relevant to PD. This review will highlight the many functions of MAMs and provide an overview of the literature that finds αSyn, in addition to several other PD-related proteins, localized there. This review will also detail the direct interaction of αSyn and αSyn-interacting partners with specific MAM-resident proteins. In addition, recent studies exploring new methods to investigate MAMs will be discussed, along with some of the controversies regarding αSyn, including its several conformations and subcellular localizations. The goal of this review is to highlight and provide insight on a domain that is incompletely understood and, from a PD perspective, highlight those complex interactions that may hold the key to understanding the pathomechanisms underlying PD, which may lead to the targeted development of new therapeutic strategies.

## 1. Introduction

### Parkinson’s Disease

Parkinson’s disease (PD) is a diverse, multi-faceted, neurodegenerative movement disorder characterized by the loss of striatum-innervating dopaminergic neurons from the substantia nigra pars compacta (SNc). The progressive degeneration of these ventral midbrain dopaminergic neurons (vmDANs) leads to the cardinal PD motor symptoms: resting tremor, bradykinesia, rigidity, and postural instability. These motor symptoms are often preceded for up to 20 years by non-motor symptoms in the prodromal stage of the disease, such as constipation or REM sleep behavior disorder (RBD) [1]. However, the origin of PD remains unknown, and there remains a lack of consensus on the initiation and spread of PD pathology (i.e., gut-first vs. brain-first), with evidence suggesting that there may be several subtypes of idiopathic PD [2].

Approximately 10–15% of PD patients have an underlying genetic heritability. Monogenic mutations in several well-established PD-associated risk (PARK) genes have identified autosomal dominant (*SNCA* [PARK 1/4], *LRRK2* [PARK 8], *VPS35* [PARK 17]) and autosomal recessive (*PRKN* [PARK 2], *PINK1* [PARK 6], *DJ-1* [PARK 7]) forms of inheritance. Moreover, several genome-wide association studies (GWAS) have identified variants in several genes associated with increased PD risk (MAPT and HLA-DRA) [3] as well as increased PD risk and progression (APOE and GBA1) [4]. Several molecular mechanisms and signaling pathways are associated with PD pathogenesis. These include defective autophagy, mitochondrial dysfunction, neuroinflammation, apoptosis, and lipid dyshomeostasis [5]. Common to these diverse molecular pathways are the localizations of several regulatory proteins found at the membrane contact sites between the ER and mitochondria, referred to as the mitochondrial-associated ER membranes (MAMs), a converging multi-functional domain acting as a molecular intersection to control several cellular functions.

## 2. Overview of Alpha-Synuclein

Central to the pathogenesis of PD are alterations to the alpha-synuclein (αSyn) protein. Polymorphisms in the *SNCA* gene that encodes αSyn result in its increased expression and are associated with sporadic PD [6,7]. Equally, rare dominantly inherited pathogenic point mutations in *SNCA* (V15A, A30P, A30G, E46K, H50Q, A53T, and A53V) are fully penetrant and lead to familial PD [8,9,10,11,12,13,14]. In addition, genomic multiplications of the *SNCA* locus, i.e., αSyn-duplication or αSyn-triplication, lead to 1.5× or 2× the respective level of the endogenous αSyn protein when compared to a single copy of the wild-type locus and are also associated with familial PD [15,16,17]. PD patients harboring a *SNCA* triplication are fully penetrant for the disease and present with an earlier onset, more aggressive clinical severity, and faster disease progression [18]. At an increasing αSyn dosage level, the native monomeric αSyn protein accumulates and aggregates in a pathological process generally accepted to be a toxic gain-of-function. The second pathological hallmark of PD, together with the degeneration of vmDANs of the SNc, is the pathological accumulation of intracellular αSyn-containing aggregates in neuronal cell bodies (i.e., Lewy bodies [LBs]) and processes (i.e., Lewy neurites [LNs]) [19] that are largely composed of lipids [20]. Importantly, the aggregation of αSyn is known to alter its properties, such as affinity to cellular membranes and membrane remodeling [21]. The αSyn–lipid interplay and the close association of αSyn and specific lipid classes in patient and cellular models of PD are further discussed elsewhere [22,23,24].

### 2.1. Structure and Conformations of αSyn

The αSyn protein natively occurs as a 140-amino acid, intrinsically disordered, monomeric protein composed of three distinct domains. The *N*-terminus (residues 1–60), which is responsible for membrane and lipid interactions, is the region where all known *SNCA* mutations associated with familial PD have been identified, which have been shown to cause pathological structural alterations to the wild-type αSyn protein. The non-amyloid-β component (NAC) domain (residues 61–95) is a hydrophobic central domain that is required for nucleation of the aggregation process [25]. The highly negatively charged *C*-terminus (residues 96–140) facilitates Ca^2+^-mediated synaptic vesicle interaction and metal ion binding [26,27].

αSyn readily binds to membranes via the KTKEGV consensus motif at the *N*-terminus, allowing αSyn to adopt an amphipathic α-helical structure that is distinct from its unbound intrinsically disordered state [28]. Once bound to membranes, αSyn has been shown to interact with free fatty acids and anionic phospholipids such as phosphatidylserine (PtdSer) [29,30], with membrane-bound αSyn found to tether vesicles together [31]. Moreover, αSyn possesses two established lipid binding motifs for glycosphingolipids (residues 34–45) and cholesterol (residues 67–78) [32,33] that can modulate the affinity of αSyn to synaptic vesicles and thereby affect membrane properties such as degree of curvature [34].

αSyn is also subject to numerous post-translational modifications (PTMs) pertinent to PD pathology [35]. For instance, >90% of αSyn detected in LBs is phosphorylated at Serine-129 (pS129-αSyn) [36,37]. While considered an indicator of pathogenesis and a driver of neurotoxicity [38], pS129-αSyn also has a physiological function in facilitating protein–protein interaction by stabilizing the *C*-terminus [39]. How pS129-αSyn transitions from having a physiological role to a pathogenic one is not clear. In addition, *C*-terminal truncations of αSyn, which is a normal cellular process, are also present in LBs and are strongly associated with its pathological aggregation [40,41].

The dogma of αSyn pathology has been to reconcile how this monomeric protein clumps into insoluble protein aggregates that comprise LBs. Several studies have provided evidence that αSyn first transitions into oligomers and then fibrils, though the mechanism of how this occurs remains not well understood [28]. Moreover, due to high structural plasticity, αSyn can access a large variety of assembly states that vary in structure, size, and morphology and which can interconvert over time [42]. To add further complexity, studies have also found that αSyn can exist physiologically as a helically folded tetramer (metastable soluble oligomers) [25,43]. While these studies primarily used cross-linking experiments to perform this work, and the notion that αSyn as a tetramer cannot be excluded, there is strong evidence and a consensus that the majority of αSyn in the cell exists as a disordered monomer [44,45]. The loss of the αSyn monomer has also been linked to the disease [46]. This concept is in part supported by evidence that low total levels of αSyn in the cerebrospinal fluid are associated with PD [47,48], but remains controversial since αSyn knockout models lack nigrostriatal PD phenotypes [49]. However, paradoxically, it is possible that increased αSyn dosage may be associated with both a toxic gain-of-function (αSyn aggregation) and a toxic loss-of-function (αSyn monomer) [46], but more evidence is required to support this. Consequently, assessing the most toxic conformational species of αSyn in terms of seeding, propagation of pathology, speed of propagation, cell dysfunction, and cell death remains highly complex and highly debated.

### 2.2. Localization of αSyn

The localization of αSyn has been long-established to be highly expressed at the presynaptic terminal of neurons in the brain, mostly as a cytosolic protein [50]. However, the subcellular localization of αSyn within the neuron has been a source of much conjecture, controversy, and debate within the field. A comprehensive study led by Przedborski and Schon used several different techniques to demonstrate that wild-type monomeric αSyn is localized to ER–mitochondria contact sites at MAM domains and not within the mitochondria, as was previously thought [51]. Since then, several other studies from different groups have also demonstrated that αSyn can be found at MAM domains [52,53,54,55]. Yet, other studies have continued to associate αSyn within the mitochondria itself [56,57,58]. As an important caveat, in many of those investigations, structurally altered αSyn was used, such as αSyn tagged to fluorescent proteins [57], carrying non-physiological quantities of exogenous αSyn [56], or higher molecular weight forms of αSyn [58]. To perhaps resolve this debate, another study led by Ma added exogenous αSyn preformed fibrils (PFFs) to a primary neuron model, with the authors finding “miniscule” evidence that physiological αSyn was bound to the mitochondria [59]. Nevertheless, the same study found that pathogenic αSyn aggregates were preferentially bound to the mitochondria [59], thus finding that pathogenic αSyn alters the normal binding and localization of the protein. In support, the pathogenic αSyn mutants (p.A30P-αSyn and p.A53T-αSyn) have been shown to cause altered membrane binding properties and mislocalization in the cell, specifically away from MAMs and towards mitochondrial membranes [29,60,61]. Despite the lack of consensus regarding the mitochondrial localization of the native monomeric αSyn protein or the lack thereof, mislocalization of αSyn and altered αSyn function at MAMs may help explain how mitochondria are morphologically and functionally impacted in PD.

αSyn has also been found at the ER [62,63,64], with the accumulation of αSyn within the ER associated with ER stress and neurodegeneration [62]. Supporting a putative αSyn-driven ER phenotype, this study led by Mazzulli found that iPS-derived neurons carrying an αSyn-triplication had fragmented ER and downstream lysosomal dysfunction [65]. αSyn has also been shown to affect ER-Golgi trafficking by binding to the small GTPase RAB1A; overexpression of RAB1A prevents Golgi apparatus fragmentation and αSyn-induced neuronal loss [66,67].

Equally debated is the localization of αSyn in the nucleus. While highly dependent on antigen retrieval methods, nuclear-localized αSyn was found using a combination of molecular techniques in the post-mortem brain of patients with dementia with LB (DLB) and neurologically normal controls, with a significantly elevated presence of the pathological pS129-αSyn in the DLB isolated nuclei [68]. As further support, αSyn has been shown to co-localize with RAN, a GTP-binding protein involved in nucleocytoplasmic transport [69], in addition to previous work suggesting importin-alpha associates with αSyn to mediate its nuclear accumulation [70]. In support of an αSyn-driven nuclear phenotype, nuclear shape defects were identified in iPS-derived DA neurons carrying pathological αSyn-p.A53T and αSyn-triplication that were not found in their respective isogenic controls [69]. This work, led by Chiba-Falek and Kantor, suggests the nuclear envelope, which serves to connect the ER to the nucleus, is a converging point of αSyn pathology [69,71]. Taken together, these aforementioned studies have provided much evidence to suggest that αSyn can be found in several organelles, with the mislocalization and accumulation of αSyn associated with PD etiology.

To further demonstrate the complexity and incomplete understanding of this fundamental PD protein, αSyn expression is not limited to neurons. In the brain, αSyn expression has been identified in microglia, oligodendrocytes, and astrocytes, albeit at lower endogenous levels than that found in neurons [72,73,74]. Additionally, αSyn is found distributed throughout the body and is expressed in diverse tissues such as the colon, lung, and skin (for review, see [75]), as well as other cell types, including erythrocytes, where it is considered one of the most abundant proteins in red blood cells [76]. This would suggest that αSyn may have a deeper and more complex multi-functional role in different conformations, subcellular localizations, and tissues that are yet to be fully revealed. αSyn has been associated with various cellular functions, the most well-established being the mobilization of synaptic vesicles and the facilitation of dopamine release [50,77]. However, αSyn has also been functionally implicated in the diverse molecular processes of lipid metabolism, innate immunity, the inflammatory response, autophagy, mitophagy, apoptosis, and Ca^2+^ homeostasis [22,78,79,80,81,82]. Importantly, the regulation of these diverse cellular processes, together with αSyn, converges and is functionally associated with MAM domains.

## 3. Mitochondria-Associated ER Membranes (MAMs)

### 3.1. Overview of the MAM

Initially designated as “Fraction X” in the seminal study by Jean Vance in 1990 [83], the MAM is a specialized domain responsible for the transfer of lipids between the ER and mitochondria. MAMs are transient intracellular domains formed in subregions of the ER in contact with mitochondria that contain enzymes with similar or higher specific activities to those found in the ER for the synthesis of lipids, namely phospholipids, triacylglycerols, cholesterol, and cholesterol esters [84]. Up to 20% of the mitochondrial surface is found in close proximity with the ER [85], with electron microscopy (EM) revealing that the ER and mitochondria are adjoined by tethers that are ~10 nm at the smooth ER and ~25 nm at the rough ER [86].

The MAM is a transient domain that is formed by local increases in unesterified (i.e., free) cholesterol in the ER. These cholesterol-rich, sphingolipid-rich assemblies known as lipid rafts facilitate highly ordered membrane microdomains that passively segregate to enrich the activity of specific MAM-resident or MAM-localized proteins [87,88]. Thus, MAM domains serve as a cellular hub, allowing protein–protein and protein–lipid interactions to transiently regulate specific signaling pathways and enzymatic activities in the cell.

### 3.2. Investigating the MAM

There are several methods to investigate the structure and function of MAM domains. The protein and lipid composition of the MAM can be assessed using several steps of sequential centrifugation, by first enriching a crude mitochondrial (CM) fraction (removing the nuclear, unbound (free) ER, and cytosolic compartments), prior to density gradient separation and high-speed centrifugation to isolate the purified MAM [83,89]. The drawbacks to subcellular fractionation experiments are the amount of starting material required, as well as the potential cross-contamination of non-MAM-localized ER, mitochondria, and other low-density membrane compartments such as lysosomes and endosomes [89].

The physical apposition of the ER and mitochondria is an established method of investigating the MAM. To investigate the structure of the MAM, EM provides excellent resolution (≤1 nm) to measure the distance between the ER and mitochondria and the length of their interaction. However, EM requires specialized equipment, may be cost prohibitive, and requires fixation, meaning live-cell imaging of MAM dynamics cannot be ascertained. Confocal microscopy utilizes the co-localization of an ER and mitochondrial protein to assess real-time ER–mitochondrial apposition, although the optical resolution of conventional confocal microscopes (~200 nm) makes data interpretation challenging. Super-resolution fluorescent microscopy that allows single-molecule localization using photoactivated localization microscopy (PALM), stochastic optical reconstruction microscopy (STORM), and stimulated emission depletion (STED) microscopy has improved resolution (≤100 nm); however, despite the technological advancements in microscopy, there remain constraints in each method [90]. To circumvent some of these limitations, new techniques to determine ER–mitochondrial apposition have been developed that use biosensors. Contact-based biosensors, such as split-green fluorescent protein (GFP)-based contact site sensors (SPLICS), can detect ER–mitochondrial apposition of either 8–10 nm or 40–50 nm, depending on the sensor used [91]. Proximity-based biosensors such as mitochondria–ER length indicator nanosensors (MERLINs) have also been developed that function without having to artificially tether the ER and mitochondria [92].

To investigate function, methods that probe specific activities of MAM-resident enzymes can be used to provide information about the MAM and thus ER–mitochondria communication. Notable MAM enzymes used to functionally characterize the MAM include the PtdSer synthases (PSS1 and PSS2) that are responsible for the de novo synthesis of PtdSer, one of the first characterized functions of the MAM [83], and the synthesis of cholesterol esters by ACAT1 [93]. Typically, these experiments involve the incorporation of radioactive lipids, performing steady-state or pulse-chase assays that can provide highly sensitive quantitative information [94]. However, there are constraints on such techniques that relate to the poor scalability and limited reproducibility of the required experimental procedures that take place over several days.

Lastly, since the ER is the primary site for intracellular Ca^2+^ storage, Ca^2+^ homeostasis at MAM domains is crucial in ER and mitochondrial communication, with the tight control of mitochondrial Ca^2+^ import essential for cellular homeostasis. To investigate Ca^2+^ homeostasis, independent measurements of Ca^2+^ stores at the ER and Ca^2+^ import into the mitochondria have been routinely performed, thereby indirectly assessing Ca^2+^ homeostasis at MAMs [53,95]. Improved developments in genetically encoded calcium indicators (GECIs), such as fluorescence resonance energy transfer (FRET) and bioluminescence resonance energy transfer (BRET), have better enabled the steady-state assessment of Ca^2+^ concentration [96,97]. Recently, MAM-specific Ca^2+^ sensors using bimolecular fluorescence complementation (BiFC) technology have been developed that allow for specific investigations of Ca^2+^ dynamics at the MAM [98].

Notwithstanding the several ways to assess MAM structure and function, the relationship between the ER–mitochondrial distance and functional activity at these contact sites remains unclear. It is important to note that since mitochondrial heterogeneity occurs, notably in neurons [99], it is reasonable to suggest that perhaps not all MAMs are also functionally equivalent.

Despite much research, the total number of proteins that either reside at the MAM or become localized to the MAM remains not fully understood. Using liquid chromatography with tandem mass spectrometry (LC-MS/MS), different studies have identified over 1000 proteins localized or enriched at the MAM in brain tissue [100,101]. Yet, only a quarter of those MAM-localized proteins in the brain were found to be conserved in the liver [101]. Whether this suggests that the MAM has differential effects in different tissues and that the same disease could lead to different MAM phenotypes in different tissues and cell types, in and outside the brain, requires further research.

### 3.3. The Functions of the MAM

**Figure 1:**
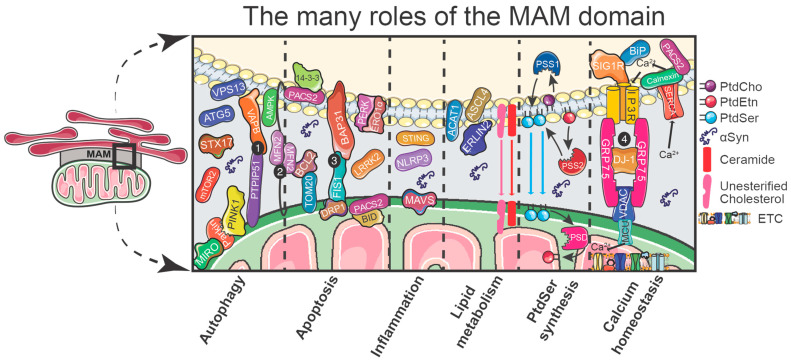
A schematic diagram containing some of the functions at the MAM domain, including relevant MAM proteins. Selected PARK proteins and PD-related proteins that have been found to localize to the MAM are shown and attributed to an associated function. These functions are loosely separated by dashed lines into six sub-sections: Autophagy, Apoptosis, Inflammation, Lipid metabolism, PtdSer synthesis, and Calcium homeostasis. Note that for each sub-section, αSyn is included, and some proteins or tethers have multiple roles or interactions other than those illustrated. The four established MAM tethers are designated by the white numbers 1–4 in the black circles: (1) VAPB-PTPIP51, (2) MFN2, (3) BAP31-FIS1, and (4) IP3R-GRP75-VDAC. Selected PARK or PD-relevant risk proteins found at the MAM in addition to αSyn include: VPS13, MIRO, Parkin, PINK1, 14-3-3, LRRK2, DJ-1, and GRP75/Mortalin. Abbreviations: VPS13: vacuolar protein sorting protein 13; ATG5: autophagy-related 5; STX17: Syntaxin 17; mTOR2: mechanistic target of rapamycin 2; MIRO: Mitochondrial Rho GTPase; PINK1: PTEN-induced putative kinase 1; VAPB: vesicle-associated membrane protein B; PTPIP51: protein tyrosine phosphatase-interacting protein-51; AMPK: 5′ AMP-activated protein kinase; MFN2: Mitofusin-2; BCL2: B-cell lymphoma 2; TOM20: translocase of the outer mitochondrial membrane complex subunit 20; PACS2: phosphofurin acidic cluster sorting protein 2; DRP1: dynamin-related protein 1; FIS1: mitochondrial fission protein 1; BAP31: B-cell receptor-associated protein 31; PERK: RNA-dependent protein kinase (PKR)-like ER kinase; ERO1α: ER oxidoreductin 1; LRRK2: leucine-rich repeat kinase 2; STING: stimulator of interferon genes; NLRP3: nucleotide-binding domain, leucine-rich repeat-containing family, pyrin domain-containing 3; MAVS: mitochondrial antiviral signaling protein; ACAT1: acyl-CoA: cholesterol acyl-transferase 1; ASCL4: acyl-CoA synthetase 4; ERLIN2: endoplasmic reticulum lipid raft-associated protein 2; PSS1: phosphatidylserine synthase 1; PSS2: phosphatidylserine synthase 2; PSD: phosphatidylserine decarboxylase; SIG1R: sigma-1 receptor; BiP/GRP78: glucose-regulated protein 78; SERCA: sarcoendoplasmic reticulum Ca^2+^ transport ATPase; IP3R: inositol 1,4,5-triphosphate receptors; GRP75: glucose-regulated protein 75; VDAC: voltage-dependent anion channel; MCU: mitochondrial calcium uniporter; PtdCho: Phosphatidylcholine; PtdEtn: Phosphatidylethanolamine; PtdSer: Phosphatidylserine; αSyn: alpha-synuclein; ETC: electron transport chain.

Vance’s pioneering work demonstrating functional roles for membrane contact sites between the ER and mitochondria has forced scientists to reconsider how organelles are studied, defined, and characterized (see review [87,102]). Thereafter, numerous studies have provided ample and compelling evidence that MAM domains are associated with several diverse molecular pathways, with important regulatory proteins required for different cellular functions either localized or enriched at MAMs (Figure 1).

### 3.4. Autophagy

Among the proteins enriched in or localized to the MAM are those involved in the initiation of autophagy. Autophagy (from the Greek words auto indicating “self” and phageîn meaning “devoured”) is a highly conserved pathway that degrades cellular components, such as protein aggregates, lipids, and defective organelles, within lysosomes. Autophagy is necessary for cellular homeostasis, and the various mechanisms and subtypes of autophagy that take place in healthy aging and disease are reviewed elsewhere [103]. Briefly, the core process of autophagy is initiated following inhibition of the mechanistic target of rapamycin (mTOR) or activation of 5′ AMP-activated protein kinase (AMPK), both of which are canonical inducers of autophagy [103], and both are localized at the MAM [104,105]. Protein Kinase B/Serine/threonine kinase (PKB/Akt), which can regulate autophagy through insulin signaling, is also found in this domain [106]. The regulation of autophagy is closely associated with the MAM since autophagosomes, the double-membrane vesicles required for autophagy, are formed at the MAM [107]. Moreover, several autophagy-related (ATG) proteins are found in this domain, including autophagy-related 5 (ATG5), autophagy-related 14 (ATG14), autophagy and beclin 1 regulator 1 (AMBRA1), and WD repeat domain phosphoinositide interacting 1 (WIP1) [107,108].

A selective subtype of autophagy is mitophagy (mitochondrial autophagy), which degrades damaged mitochondria in order to maintain mitochondrial function. Key mediators of mitophagy include PTEN-induced putative kinase 1 (PINK1), a serine/threonine kinase, and Parkin, an E3 ubiquitin ligase [109,110]. The mechanisms by which PINK1–Parkin-mediated mitophagy takes place are reviewed elsewhere [111], though in brief, PINK1 recruits Parkin to clear damaged mitochondria [112]. Defective mitophagy has been strongly associated with PD since mutations in *PRKN (PARK2)*, which encodes Parkin, and *PINK1* (PARK6) are causative for autosomal-recessive familial PD [113,114].

Recently, there have been several studies associating mitophagy with the MAM, with MAM localization common to several subtypes of mitophagy. During canonical mitophagy, PINK1 relocalizes to the MAM and associates with MAM-localized BECN1 to promote the enhancement of ER–mitochondrial contact sites and the formation of omegasomes, precursors to autophagosomes [115]. Beclin1 (BECN1), a key component of the class III phosphatidylinositol 3-kinase (PtdIns3K) complex (containing BECN1, ATG14, vacuolar protein sorting (VPS)34/PIK3C3, and VPS15/PIK3R4), is also localized at the MAM and can recruit Parkin for mitophagy in a PINK1-independent process, with Parkin enhanced at the MAM following the induction of mitophagy [115,116]. Independent of Parkin, an alternative E3 ubiquitin ligase, Glycoprotein 78 (GP78), can also be recruited to the MAM to regulate mitophagy [117,118], and furthermore, regulators for alternative mitophagy pathways such as FUN14 domain containing 1 (FUNDC1) that facilitate receptor-mediated mitophagy are also found at the MAM [119,120].

In addition to mitophagy, several proteins required for the maintenance of mitochondrial function and mitochondrial dynamics are localized at the MAM, such as voltage-dependent anion channel 2 (VDAC2) [121] and dynamin-related protein 1/dynamin-like protein 1 (DRP1/DLP1) [122]. The outer-mitochondrial membrane (OMM) protein, mitochondrial fission protein 1 (FIS1), that recruits DRP1 to the OMM stimulates mitochondrial fission [123], with mitochondrial fission also occurring during the early stages of cell death [124]. However, once FIS1 is organized at the MAM, it has an alternative non-fission role. FIS1 has been found to interact with proteins associated with autophagy, including the SNARE protein Syntaxin 17 (STX17) [125], and apoptosis, such as B-cell receptor-associated protein 31 (BAP31), forming an integral MAM tether [126] (see Section 4.2.1). Interestingly, FIS1 has a functional role at the membrane contact sites of other organelles, such as the peroxisome and lysosome, and is reviewed elsewhere [127].

### 3.5. Apoptosis

MAM proteins that are associated with apoptosis often include one or many of the aforementioned proteins that, often through Ca^2+^ signaling, regulate the MAM, such as BAP31, which can recruit the targeted apoptotic protein pro-caspase 8 [126,128]. Additionally, local increases in specific lipids, such as ceramide, can initiate apoptosis at the MAM by binding VDAC2 [129]. PERK (RNA-dependent protein kinase (PKR)-like ER kinase), a key ER stress sensor of the unfolded protein response (UPR), is enriched at the MAM domain and has a functional role in maintaining ER–mitochondrial apposition [130]. PERK contributes to apoptosis by facilitating the pro-apoptotic C/EBP homologous protein (CHOP) and through the propagation of reactive oxygen species (ROS) between the ER and the mitochondria [130]. Recent studies have also found that PERK directly binds with the oxidative stress response (ER oxidoreductin 1 (ERO1α)), forming a complex at the MAM, oxidizing several MAM proteins, and regulating mitochondrial dynamics [131]. Moreover, the PERK–ERO1α interaction facilitates Ca^2+^ transfer in both ER and mitochondria while limiting oxidative stress [131]. In response to apoptotic inducers, the multi-functional sorting protein phosphofurin acidic cluster sorting protein 2 (PACS2) translocates Bid, a pro-apoptotic member of the B-cell lymphoma 2 (BCL2) protein family [132], to the mitochondria to initiate a series of cascades that conclude with the release of cytochrome C and activation of caspase-3, thus causing cell death [131].

### 3.6. Inflammation

In immune cells, such as microglia and macrophages, the nucleotide-binding domain, leucine-rich repeat-containing family, and pyrin domain-containing 3 (NLRP3) inflammasome are a multiprotein complex that is recruited to and is in part activated at the MAM via cholesterol trafficking [133,134,135]. Mitochondrial antiviral signaling protein (MAVS), which serves as an important platform for cellular antiviral response in innate immunity, is also localized at the MAM, which is in part recruited by the MAM-resident protein GP78 [136,137]. In addition, stimulator of interferon genes (STING), a key regulator of innate immunity, has been shown to be present at the MAM and is known to bind MAVS to increase the interferon response to viral infection [138]. It is increasingly recognized that chronic neuroinflammation is causally relevant to PD. The chronic activation of STING causes neuroinflammation and degeneration of dopaminergic neurons [139]. Tying together pathogenic αSyn to neuroinflammation, αSyn aggregates increase the expression of STING, with STING-deficient mice protected from dopaminergic neuronal loss [140].

### 3.7. Lipid Metabolism

As identified by Vance and colleagues, the MAM is critical for the regulation of lipid metabolism and transport, with several proteins regulating lipid metabolism and transport being enriched at the MAM. This includes enzymes involved in cholesterol metabolism (acyl-CoA: cholesterol acyl-transferase 1 (ACAT1) [84], and endoplasmic reticulum lipid raft-associated protein (ERLIN2)) [141], the synthesis of triglycerides (acyl-CoA: diacylglycerol acyltransferase 2 (DGAT2)) [142], sphingolipid metabolism (serine palmitoyltransferase (SPTLC1) and sphingolipid desaturase (DEGS1) [143,144]), and the recruitment and modulation of long- and very long-chain fatty acyl-CoA ligases using long-chain acyl-CoA synthetases (ACSL). ACSL1 is responsible for the activation of monounsaturated fatty acids (MUFAs), and ACSL4 and ACSL6 are responsible for the activation of polyunsaturated fatty acids (PUFAs). ASCL4 is also one of the key enzymes required for ferroptosis, an iron-dependent measure of cell death [145], which is linked with PD and αSyn dosage [146]. At the MAM, the biosynthesis of MUFAs from saturated fatty acids occurs via stearoyl-CoA desaturase 1 (SCD1) [147], while the ubiquitin-X domain adaptor 8 (UBXD8) regulates the composition and saturation of fatty acids [148].

Thus, the MAM simultaneously regulates the generation, oxidation, elongation, and desaturation of fatty acids, as well as the transport and saturation of phospholipids [149]. Moreover, alterations to the fatty acid chain length and degree of saturation can alter both the biochemical and biophysical properties of lipid membranes, such as thickness, permeability, curvature, and fluidity, and are therefore tightly regulated by MAM domains [148,150].

### 3.8. Phosphatidylserine Synthesis

One of the first established functional pathways specific to the MAM is the de novo synthesis of phosphatidylserine (PtdSer), an essential anionic phospholipid required for the structure and function of cell membranes [83,84]. The biosynthesis of PtdSer takes place using two evolutionally conserved MAM-localized enzymes, PtdSer Synthase 1 (PSS1) and PtdSer Synthase 2 (PSS2) [151]. As substrates for these reactions, the PSS enzymes use glycerophospholipids, phosphatidylcholine (PtdCho), and phosphatidylethanolamine (PtdEtn), which are synthesized in the ER via the CDP-choline and CDP-ethanolamine pathways, respectively, commonly referred to as the two branches of the Kennedy pathway [151,152]. Briefly, acting as base-exchange enzymes, PSS1 preferentially uses PtdCho to generate PtdSer by catalyzing the choline headgroup for serine, whereas PSS2 exclusively uses PtdEtn to generate PtdSer with serine replacing the ethanolamine (Figure 1) [83,84]. The exclusivity for PSS1 to use PtdCho to generate PtdSer is debated within the field, since under specific experimental conditions, PSS1 generated PtdSer from PtdEtn (see review [153]). The final step in the PtdSer pathway is its decarboxylation to PtdEtn. This process takes place following the transport of PtdSer into the inner mitochondrial membrane, a process that requires ATP [154]. Once in the mitochondria, PtdSer is rapidly decarboxylated via phosphatidylserine decarboxylase (PSD) [151].

Importantly, PtdSer metabolism has relevance to PD. At the MAM, reduced de novo PtdSer synthesis was found in αSyn models carrying familial SNCA mutations p.A30P and p.A53T [61], and αSyn readily binds membranes rich in PtdSer [60,155]. After αSyn is recruited to these PtdSer-rich membranes, it undergoes conformational changes, though the functional consequences of this are unclear [156]. The distribution, dynamics, and functional roles of PtdSer within the cell are diverse, but the majority of the PtdSer within the cell is found at the plasma membrane, specifically the inner leaflet [157]. Relevant to PD, PtdSer has notable roles in autophagy and inflammation. At the MAM, PtdSer, together with PtdEtn, binds to LC3/ATG8 to facilitate the formation of autophagosomes [158]. Away from the MAM, loss of PtdSer asymmetry at the plasma membrane and PtdSer externalization is an early indicator of apoptosis and the “eat-me” signal for cells of the immune system, such as T-cells and microglia, to phagocytose both dying and viable neurons [159], with infiltration of T-cells and microglia found in the SNc of PD patients [160,161]. Together, this links MAM-generated PtdSer to PD neuroinflammation, yet how PtdSer synthesis at the MAM relates to its externalized PtdSer remains unclear.

### 3.9. Calcium Homeostasis

Calcium signaling is integral to MAM homeostasis, and several proteins that are enriched or localized at the MAM include those involved in the regulation of calcium, notably sarcoendoplasmic reticulum (SR) Ca^2+^ transport ATPase (SERCA) 2b, which regulates Ca^2+^ influx [162], and inositol 1,4,5-triphosphate receptors (IP3R), which mediate Ca^2+^ efflux [163]. Chaperones and oxidoreductases that relocalize to MAMs contribute to ER–mitochondria Ca^2+^ homeostasis, such as the chaperones sigma-1 receptors (SIG1R) [164], calnexin [165], and the oxidoreductase ERO1α [131]. The palmitoylation of calnexin acts as a molecular switch, facilitating its regulation of Ca^2+^ signaling as opposed to protein folding [162]. At the MAM, the distribution of calnexin occurs by PACS2, with calnexin interacting with and regulating SERCA2b [162,165]. The regulation of Ca^2+^ across the MAM is facilitated by the MAM tether IP3R-GRP75-VDAC1 (see Section 4.2.3). Ca^2+^ signaling across the ER, MAM, cytosol, and mitochondria is comprehensively reviewed elsewhere (see [166,167]).

## 4. Regulating ER–Mitochondrial Apposition

### 4.1. PACS2

Amongst the established proteins that regulate ER–mitochondria interactions, PACS2 is particularly notable as it is evidenced to control the ER–mitochondrial axis and regulate ER–mitochondria apposition [168]. At both the ER-facing and mitochondria-facing membranes, PACS2 interacts with and organizes numerous proteins enriched at the MAM [165,168,169]. Depletion of PACS2 alters MAM integrity via BAP31-dependent mitochondrial fragmentation and ER uncoupling via altered Ca^2+^ signaling [168], with overexpression of PACS2 found to rescue MAM integrity [105]. PACS2 expression has been found to be upregulated in the AD brain at post-mortem and is also associated with insulin resistance, which in turn is linked with PD progression [170,171]. The underlying mechanisms of PACS2 require further understanding and are discussed elsewhere [170].

PACS2, together with the four established MAM tethers (FIS1-BAP31, PTPIP51-VAPB, IP3R-GRP75-VDAC, and MFN2), helps stabilize and regulate the interaction of the ER and mitochondria. Alterations in the activities of these structural tethers and lipid-sensing proteins regulate the formation and dispersion of MAM domains and may be associated with multiple signaling pathways and cellular functions.

### 4.2. MAM Tethers

#### 4.2.1. FIS1-BAP31

At the MAM, FIS1 interacts and forms a MAM tether with an integral ER membrane chaperone, BAP31. This FIS1–BAP31 tether recruits and activates pro-caspase 8 that cleaves BAP31 into the pro-apoptotic p20BAP31 and transmits apoptotic signals from the mitochondria to the ER [126]. This pro-apoptotic signal then returns to the ER from the mitochondria via Ca^2+^ release from ER stores, activating and opening the mitochondrial permeability transition pore (mPTP), with prolonged opening of the mPTP leading to cell death [126,172]. In immune cells, BAP31 is involved in the neuroinflammatory process via CD11/CD18 association, T cell activation, and interplay with major histocompatibility complex (MHC) class I molecules [173,174,175]. Interestingly, deficiency of BAP31 has been found to contribute to the formation of amyloid-beta plaques in Alzheimer’s disease (AD) [176]. Furthermore, with particular relevance to PD, BAP31 negatively regulates the expression of monoamine oxidase A (MAO-A), the enzyme that degrades monoamine neurotransmitters, such as dopamine, via cell division cycle-associated 7-like (R1/RAM2/CDCA7L/JPO2, a transcriptional repressor of MAOA) [177].

#### 4.2.2. PTPIP51-VAPB

The integral ER protein, vesicle-associated membrane protein B (VAPB) forms a well-established tethering complex with the protein tyrosine phosphatase-interacting protein-51 (PTPIP51) located in the OMM [178]. Subdomains within VAPB have been shown to facilitate ER membrane curvature [179]. The dissociation of VAPB is triggered by p97/Valosin-containing protein (VCP) via the negative regulation of VPS13D, a homolog of VPS13C (PARK23), associated with early-onset autosomal-recessive PD [180,181]. The VPS13 proteins are phospholipid transporters that facilitate ER-phagy by packing ER into autophagosomes [182]. Suppression of VPS13D leads to extensive ER–mitochondrial tethering [183]. PTPIP51 is a carrier for negatively charged phospholipids, including PtdSer and phosphatidic acid (PA). PTPIP51 is also required during the biogenesis of lipid droplets, which is regulated at the MAM by the oxysterol-binding related proteins (ORP) 5/8 complex [184]. Overexpression of PTPIP51 increases ER–mitochondrial contact sites, and reciprocally, knockdown reduces these ER–mitochondrial connections [178]. PTPIP51B has also been shown to mediate the concentration of mitochondrial cardiolipin [185]. These tethering proteins, found in the neuronal synapse, have been implicated in the regulation of autophagy, synaptic activity, and Ca^2+^ exchange [52,186,187] and have been associated with neurodegenerative diseases including PD, frontotemporal dementia (FTD), and amyotrophic lateral sclerosis (ALS) [178,186,187].

#### 4.2.3. IP3R-GRP75-VDAC1

An established functional role at the MAM is the regulation of Ca^2+^ through the tripartite complex/tether of inositol 1,4,5-trisphosphate receptors (IP3R), glucose-regulated protein 75 (GRP75), and voltage-dependent anion channel 1 (VDAC1). IP3R is an ER Ca^2+^ channel that, together with VDAC1 in the OMM, recruits GRP75 to mediate the transfer of Ca^2+^ between the ER and mitochondria, with the mitochondrial calcium uniporter (MCU) transporting Ca^2+^ into the mitochondrial matrix [188]. The PARK protein DJ-1, which is a protein deglycase, physically interacts with and regulates the IP3R-GRP75-VDAC complex [189]. Other proteins that have co-regulatory roles in this tripartite complex include the type II ER membrane protein SIG1R, which forms a Ca^2+^-sensitive chaperone complex with glucose-related protein 78 (GRP78/BiP), interacting and modulating IP3R [164,190]. Upon activation of the IP3Rs, there is a decrease in Ca^2+^ concentration at the MAM that is taken up by the mitochondria. The decrease in Ca^2+^ concentration at the MAM redistributes SIG1Rs away from the MAM, causing the dissociation of the GRP78/BiP-SIG1R complex [164].

#### 4.2.4. MFN2

Mitofusin 2 (MFN2) is a mitochondrial fusion protein that, together with MFN1, coordinates the fusion of the OMM of adjacent mitochondria [191]. In addition, MFN2 is a MAM protein that tethers the ER and mitochondria and serves as a suppressor of mitophagy [192,193]. The ubiquitination of MFN2 by Parkin in a PINK1-dependent mechanism regulates MAM integrity by the destruction of ER–mitochondria contact sites, with MFN2 disassembly triggered by p97/VCP [193]. At the MAM, MFN2 is directly regulated by AMPK, a cellular energy sensor. In states of energy deprivation, AMPK is activated and translocates from the cytosol to the MAM, directly interacting with MFN2 to induce autophagy and increase the formation of MAMs [104].

## 5. Direct Association of αSyn with Proteins at the MAM

αSyn has been found at the MAM in different studies [52,53,55,61]. Increasing levels of αSyn have been shown to increase the number of ER–mitochondria contact sites and consequently increase mitochondrial Ca^2+^ uptake from the ER [53]. In contrast, pathogenic point mutations in αSyn have been shown to reduce ER–mitochondria apposition, communication, and MAM activity [61]. In addition, there are several studies detailing that αSyn binds or associates with numerous proteins at MAMs (Table 1).

During the formation of the MAM, there is an enrichment of several ER-resident chaperones that either directly bind to αSyn, including BiP/GRP74 and GRP94 [100,194,196], or associate with αSyn, such as calnexin [194,195]. These proteins are involved in multiple processes ranging from protein folding, Ca^2+^ signaling, the ER stress response, quality control, and ER-associated degradation (ERAD) [204]. Unlike GRP78, GRP94 has an additional role in the initiation of the immune response, with GRP94 client proteins including MHC class I [197,205]. Calnexin both associates with αSyn and, in the ER, interacts with FAM134B to facilitate ER-phagy as a mechanism to degrade αSyn [165,194,195]. In PD patients, ER-phagy receptors FAM134B and CCPG1 are reduced, suggesting PD pathogenesis may be associated with deficient ER-phagy [195]. Indeed, overexpression of FAM134B in PD mice enhanced ER-phagy and led to neuroprotective effects by increasing autophagy and reducing αSyn levels [195].

The predominant SERCA pumps found in neurons in the brain are SERCA2 pumps (see reviews [206,207]), which have been evidenced to be regulated by calnexin at the MAM [162]. Notably, aggregates of αSyn, but not the monomeric protein, were also found to directly bind to SERCA pumps and stimulate their activity, leading to the release or uptake of ER Ca^2+^ [208]. In other PD models, alterations in SERCA activity were associated with the loss of the DJ-1 protein [209], with the familial PD mutation in leucine-rich repeat kinase 2 (LRRK2)-p.G2019S directly interacting with and modifying SERCA [210]. In these studies, the restoration of SERCA activity to normal levels was neuroprotective [208,209,210]. Importantly, these studies have revealed that pathogenic structures (aggregate/mutant vs. monomeric protein) have different physiological effects, impacting Ca^2+^ regulation. This suggests that mitigating Ca^2+^ dyshomeostasis at MAMs could be a therapeutic strategy that warrants further investigation.

Native monomeric αSyn binds to the VAPB at the MAM, as evidenced by immunoprecipitation and proximity ligation assays (PLAs), with pathogenic αSyn (αSyn-A30P and αSyn-A53T) found to increase VAPB binding compared to the native αSyn protein [52]. The consequence of the pathogenic αSyn disrupting the VAPB–PTPIP51 tether was the dysregulation of Ca^2+^ homeostasis by reducing IP3R-mediated Ca^2+^ delivery to the mitochondria [52]. This reduction in mitochondrial Ca^2+^ import is associated with reduced mitochondrial ATP production [52]. Tau, encoded by the familial AD gene *MAPT* that is also linked with increasing PD risk [3], also associates with VAPB, and in cases of abnormal tau (depletion or mutation of the protein), ER–mitochondria apposition decreases as mitochondrial cholesterol import is impaired [211]. Restoring the VAPB–PTPIP51 tether and thereby restoring MAM served to counterbalance the abnormal tau [211]. This convergence of tau and αSyn on VAPB at the MAM may indicate a molecular crosstalk between these distinct diseases, highlighting the therapeutic potential of targeting VAPB and the role of the MAM as a converging neurodegenerative domain.

Studies have also shown that αSyn binds proteins at the OMM that perform a regulatory role at the MAM domain, including the translocase of the outer mitochondrial membrane complex subunit 20 (TOM20) [198], which facilitates apoptosis by mediating the transfer of BCL2, a key protein regulator of apoptosis from the ER, to the MAM [212]. Single-molecule electrophysiology studies have found that the *C*-terminus of αSyn directly binds to VDAC and is a potent regulator of mitochondrial Ca^2+^ signaling [55]. The *C*-terminus of αSyn facilitates a “charge inversion” of VDAC, allowing this Ca^2+^ channel to be a cation at low Ca^2+^ concentrations and an anion at higher concentrations [55]. However, the extent of VDAC charge inversion and its association with PD pathology remain incompletely understood.

At the mitochondria, PARK proteins PINK1–Parkin and LRRK2 mediate the removal of clusters of Mitochondrial Rho GTPase (MIRO) to facilitate the clearance of damaged mitochondria. MIRO is localized on the OMM and regulates ER–mitochondria contact sites by serving as a cytosolic Ca^2+^ sensor [213,214]. αSyn, specifically at the *N*-terminus, functions independently of the PINK1–Parkin or LRRK2 pathways to directly bind to MIRO and form a complex at the OMM, allowing MIRO to accumulate and delay mitophagy [80]. The association of MIRO1 with PD is strengthened by evidence that rare variants in *RHOT1*, the gene that encodes MIRO, increase PD risk [215]. Mortalin (glucose-regulated protein 75 (GRP75)) is a molecular chaperone belonging to the heat shock protein 70 (HSP70) family and is another direct αSyn binding partner that is enriched at the MAM domain [194]. Mortalin negatively correlates with αSyn accumulation, and the loss of Mortalin is associated with PD progression and AD pathology, suggesting Mortalin loss may be a general feature of neurodegeneration [216]. The multi-functional DJ-1 protein directly interacts with Mortalin and, through this interaction, regulates the integrity and function of the MAM [217]. DJ-1 also serves as an antioxidant and regulates PINK1/Parkin-mediated mitophagy via activation of Akt. While DJ-1 and αSyn do not directly interact, they are indirectly associated through a number of protein partners [194]. Therefore, as evidenced in many disparate studies, in addition to αSyn, several PD risk proteins are found at the MAM and could be consequential to the underlying pathomechanism of disease.

### 5.1. Association of αSyn Interacting Proteins at the MAM

There is also evidence for indirect involvement of αSyn at the MAM mediated by confirmed interacting proteins of αSyn, including the 14-3-3 proteins and synphilin-1. The 14-3-3 proteins are a family of highly conserved adaptor proteins with at least seven mammalian isoforms. The 14-3-3 proteins are kinase-dependent activators of tyrosine hydroxylase (TH), the rate-limiting enzyme for the biosynthesis of dopamine [218], and are confirmed physical interaction partners of αSyn, with the proteins sharing considerable homology [219]. The link to PD pathology is strengthened by evidence that LRRK2 can bind to 14-3-3 protein isoforms via its phosphorylation sites, that 14-3-3 proteins are regulated by LRRK2 kinase activity [220], and that overexpression of theta isoform (14-3-3θ) mitigates LRRK2- and αSyn-mediated toxicity [202,221]. At the MAM, the 14-3-3 proteins are physical interaction partners of PACS2 [169] and are regarded as regulators of autophagy [222]. Additionally, 14-3-3 proteins are bound to HS1 binding protein 3 (HS1BP3) at MAMs, with HS1BP3 considered a negative regulator of autophagy [200,201]. Strengthening the association with movement disorders, variations in the *HS1-BP3* gene are associated with essential tremor [223], with 14-3-3 proteins being major constituents of LBs in PD and diffuse Lewy Body disease (DLBD) and suggested to play a role in LB formation [224].

Synphilin-1 is another αSyn-interacting protein that is also a main constituent of LBs. While normally a cytosolic protein, synphilin-1 can directly bind PINK1 on the OMM and become ubiquitinated by Parkin [225]. The maturation of synphilin-1 is also regulated by sphingolipids [226], a primary component of MAM domains. In mitochondria, synphilin-1 can additionally combine with PINK1 to promote the Parkin-independent mitophagy pathway [203]. Synphilin-1 also interacts with two other proteins found at the MAM, LRRK2 and AMPK, though the precise subcellular localization of this interaction is unclear [227,228].

### 5.2. Altering αSyn-Interacting Proteins at the MAM

Considering that αSyn interacts with several proteins at the MAM and that those interactions may be pertinent to PD pathology (Table 1), research is ongoing to alter those aforementioned αSyn–MAM protein interactions. Additionally, by interacting with αSyn, those proteins, which may themselves have additional interacting partners, could influence the regulation of other proteins. For instance, αSyn is known to interact with and regulate VDAC [55], and a notable interacting partner of VDAC is hexokinase 2 (HK2), forming the VDAC-HK2 complex [229]. HK2 has also been found at the MAM and serves to regulate glucose metabolism under Akt/mTOR [105,230]. In the absence of glucose, HK2 inhibits mTOR, facilitating autophagy [231]. Associating HK2 and αSyn, a study by Rajendran et al., found that a small membrane-binding peptide of HK2 (HK2pep) prevented the association of αSyn with VDAC [232]. While further studies are required, it suggests that αSyn, glucose metabolism, and autophagy are linked and associated at MAMs and could be relevant in understanding the underlying pathomechanisms of PD.

In contrast to reducing αSyn–MAM protein interactions, a separate study has found that increasing the association of αSyn with GRP78/BiP can reduce αSyn toxicity [196]. In support of this, GRP78/BiP is known to decline during normal aging, and reducing GRP78/BiP in animal models is associated with increased αSyn toxicity [233]. Several studies have used GRP78/BiP as a therapeutic target in models of PD (reviewed [234]), although how these small molecules affect the GRP78/BiP–αSyn interaction remains unclear.

### 5.3. αSyn as a MAM Regulator?

**Figure 2:**
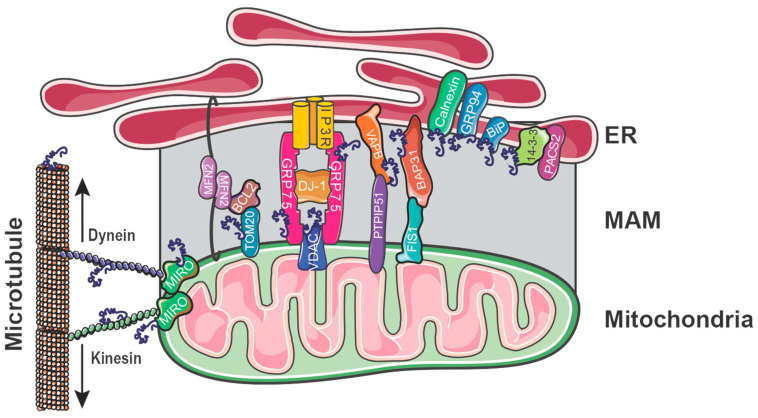
αSyn binding partners associated with the MAM. αSyn has been shown to be directly bound to MAM tethers VAPB-PTPIP51, BAP31-FIS1, and IP3R-GRP75-VDAC and can be indirectly bound to MFN2. Selected proteins at the ER-facing and mitochondria-facing MAM domains are shown. Note that the illustration of αSyn–protein binding at MAMs is for illustrative purposes and does not indicate which domain of αSyn (*N*-, *NAC*-, *C*-terminus) is bound. Abbreviations: MIRO: Mitochondrial Rho GTPase; MFN2: Mitofusin-2; BCL2: B-cell lymphoma 2; TOM20: translocase of the outer mitochondrial membrane complex subunit 20; IP3R: inositol 1,4,5-triphosphate receptors; GRP75: glucose-regulated protein 75; VDAC: voltage-dependent anion channel; VAPB: vesicle-associated membrane protein B; PTPIP51: protein tyrosine phosphatase-interacting protein-51; FIS1: mitochondrial fission protein 1; BAP31: B-cell receptor-associated protein 31; GRP94: glucose-regulated protein 94; BiP/GRP78: glucose-regulated protein 78; PACS2: phosphofurin acidic cluster sorting protein 2.

A recent study by Ramezani and colleagues found that αSyn has a tight “dual-anchor” attachment to the ER and mitochondria and could itself regulate the MAM by tightening and loosening ER–mitochondria contact sites [53]. Using small unilamellar vesicles (SUVs) as a model to mimic the composition and curvature of synaptic vesicles, this work led by Eliezer and Baird proposed that αSyn can function as a double-anchor, binding two synaptic vesicles 50 nm across and thereby tightening their interaction [31,235]. At the MAM, which has an apposition of <50 nm, it is possible that this same double-anchor mechanism may also take place. In support, there are numerous studies that find αSyn binds to integral MAM tethers, in addition to ER-facing and mitochondria-facing proteins at the MAM (Figure 2).

Facing the ER, αSyn is a confirmed binding partner for both VAPB and BAP31 [52,236,237], serving both the PTPIP51-VAPB and FIS1-BAP31 tethers, respectively. Additionally, αSyn is a confirmed binding partner of integral ER-resident proteins BiP, calnexin, and GRP94 that are enriched during the formation of the MAM [100,194,195,196]. At the OMM, αSyn has confirmed binding partners in MIRO, TOM20, and VDAC [55,80,198]. αSyn is also a binding partner of GRP75/Mortalin [194], thus associating to both VDAC and GRP75, αSyn regulates two components in the IP3R–GRP75–VDAC tether. There is also evidence to suggest that the remaining MAM tether, MFN2, is integrated with the proteins of the BCL2 family [238]. Considering that recent work has identified a role for TOM20 in mediating the transfer of BCL2 from the ER to mitochondria across the MAM [212] and that TOM20 is an αSyn binding partner [198], there is reasonable evidence to suggest that αSyn, either directly or indirectly, controls all four of the MAM tethers and is thus able to regulate ER–mitochondria apposition, possibly aided by MIRO1, via its association to adaptor proteins and microtubules [213]. To support this notion, αSyn is also known to interact with the molecular motors kinesin-1 and dynein and is thus implicated in microtubule transport [239]. Moreover, αSyn has been evidenced as a microtubule dynamase directly associating with α-tubulin, with pathogenic αSyn mutations compromising this αSyn/tubulin interaction [240,241]. Thus, there are many studies providing strong evidence that αSyn plays a substantial regulatory role in the dynamics at the MAM domain and at the MAM itself. Yet, how αSyn orchestrates this transient domain to contribute to the regulation of these MAM-enriched proteins and lipids and how they cooperate together requires further research and understanding, with the precise mechanisms yet to be elucidated.

## 6. Conclusions and Future Directions

It is clear that αSyn is inexorably entwined in PD. The greater understanding of both the disease and the protein that is most implicated in its pathology has revealed that PD and αSyn are both aligned in their complexity. Recent clarification of the functional roles of membrane contact sites and MAM domains has led to the understanding that these ER–mitochondria contact sites are physiologically important and serve as a convergence point and platform for diverse cellular mechanisms. Several studies have provided compelling evidence that a dysfunctional MAM domain could be particularly relevant in the pathogenesis of PD, with several PD-associated risk proteins, in addition to αSyn, localized or enriched there.

The functional consequence of αSyn binding to the MAM is that it can alter, directly or indirectly, one or several of the MAM functions (autophagy, apoptosis, inflammation, lipid metabolism, de novo synthesis of PtdSer, and Ca^2+^ homeostasis). Moreover, there remain several unanswered questions, both in regard to the mechanisms of the MAM and the interplay of αSyn. For instance, how does the increased abundance of αSyn enriched at MAM domains [61] alter MAM function(s)? Indeed, are all MAMs equal, or can they be specialized? Is there a hierarchy to these functional alterations, and do these alterations occur in the same direction? Equally, how does the increased abundance of αSyn at MAM domains alter the ER–mitochondrial dynamics? Does the altered number of connections affect the material transferred across the MAM, or does the amount of material transferred across the MAM alter the number of ER–mitochondrial connections? Moreover, given that the MAM is a transient domain, the temporal effect remains rather incompletely understood. How long do these connections last? How do they detach, and do the different distances identified from the ER and mitochondria apposition studies relate to altered function?

Thus, due to these open questions, it is clear that more research is needed to further decipher MAM function and how αSyn–MAM interactions might be particularly important to PD. Therefore, considering that PD requires much-needed therapeutic intervention strategies, the discovery of new targets and subsequent restoration of MAM function(s) by pharmacological intervention could be an innovative approach to facilitating the development of targeted treatments against PD and the α-synucleinopathies.

## Figures and Tables

**Table 1:** List of αSyn interacting partners at the MAM.

Role(s) at the MAM	Location	Association with PD Pathology	References
Associates to Calnexin	ER-facing	Increases ER stress Reduces ER-phagy (in ER)	[194,195]
Binds to GRP78/BiP	ER-facing	Increases ER stress Activates unfolded protein response (UPR)	[196]
Binds to GRP94	ER-facing	Alters Ca^2+^ homeostasis Alters MHC class I expression	[65,100,194,197]
Binds to VAPB	ER-facing Part of VAPB-PTIP51 tether	Alters Ca^2+^ homeostasis Reduces mitochondrial ATP production	[52,187]
Binds to TOM20	OMM-facing	Inhibits mitochondrial protein import	[198]
Binds to VDAC	OMM-facing Part of IP3R-GRP75-VDAC complex	Alters mitochondrial Ca^2+^ signaling	[55]
Associates with GRP75 (Mortalin)	Between ER and MAM Part of IP3R-GRP75-VDAC complex	Impairs mitochondrial function Aggregated αSyn decreases Mortalin	[194,199]
Binds to MIRO1	OMM-facing	Accumulation of MIRO1 Delays mitophagy	[80]
Binds to 14-3-3	14-3-3 interacts with PACS2 14-3-3 interacts with HS1BP3	Increases αSyn aggregation Alters autophagy	[200,201,202]
Binds to Synphilin-1	Synphilin-1 interacts with PINK1/Parkin	Alters mitophagy	[203]
